# Association between outpatient follow-up and incidence of revision after knee and hip replacements: a population-based cohort study

**DOI:** 10.1186/s12891-023-06190-7

**Published:** 2023-02-08

**Authors:** Rafael Pinedo-Villanueva, Spyros Kolovos, Edward Burn, Antonella Delmestri, Lindsay K. Smith, Andrew Judge, Sarah R. Kingsbury, Martin H. Stone, Philip G. Conaghan

**Affiliations:** 1 Nuffield Department of Orthopaedics, Rheumatology and Musculoskeletal Sciences, University of Oxford, Old Road, Oxford, OX3 7LD UK; 2 Faculty of Health and Applied Sciences, University of the West of England, Rm 2G40 Glenside Campus, Blackberry Hill, Bristol, BS16 1DD UK; 3 Translational Health Sciences, Bristol Medical School, University of Bristol, Learning and Research Building, Level 1, Southmead Hospital, Bristol, BS10 5NB UK; 4 Leeds Institute of Rheumatic and Musculoskeletal Medicine (LIRMM), University of Leeds, Chapel Allerton Hospital, Chapeltown Road, Leeds, LS7 4SA UK; 5 NIHR Leeds Biomedical Research Centre, Leeds, UK

**Keywords:** Osteoarthritis, Joint replacement surgery, Follow-up, Revision surgery, Mortality, Hip, Knee

## Abstract

**Background:**

Follow-up visits 5 or 7 years after surgery were recommended for people having primary hip or knee replacement. The benefits of this practice to patients and the healthcare system, however, have not yet been specifically examined. The aim of this study was to investigate the association between long-term follow-up outpatient hospital visits and revision rates for patients who undergo primary knee or hip replacement surgery.

**Methods:**

Cohorts were identified for patients undergoing knee or hip replacement surgery using medical records from primary care practices within the UK Clinical Practice Research Datalink (CPRD) GOLD dataset linked to hospital records from the English Hospital Episodes Statistics (HES) data. Two groups of patients were compared in terms of revision and mortality rates: those with at least one long-term (between five and 10 years since primary surgery) follow-up visit at the orthopaedic department (‘Follow-up’ group), and those without (‘No follow-up’ group).

**Results:**

A total of 9856 (4349 in the Follow-up group) patients with knee replacement and 10,837 (4870 in the Follow-up group) with hip replacement were included in the analysis. For knee replacement, the incidence of revision was 3.6% for those followed-up and 0.6% for those not followed-up. An adjusted regression model confirmed the difference in the hazard ratio (HR) for revision was statistically significant (HR: 5.65 [95% CI 3.62 to 8.81]). Mortality at 4 years was lower for the Follow-up (17%) compared to the No follow-up group (21%), but this difference was not statistically significant (HR: 0.95 [0.84 to 1.07]). For hip replacement, the incidence of revision rates were 3.2 and 1.4% for the follow-up and not follow-up groups, respectively, the difference being statistically significant (HR: 2.34 [1.71 to 3.20]). Mortality was lower for the Follow-up (15%) compared to the No follow-up group (21%), but the difference was not statistically significant (HR: 0.91 [0.81 to 1.02]).

**Conclusion:**

Patients attending follow-up orthopaedic consultations show a higher risk of revision surgery compared to those who are not followed-up. A cause for this difference could not be identified in this study but a likely explanation is that surgeons play an effective role as ultimate arbitrators when identifying patients to be included in long-term follow-up lists.

**Supplementary Information:**

The online version contains supplementary material available at 10.1186/s12891-023-06190-7.

## Introduction

The replacement of a damaged knee or hip joint with an artificial one constitutes a common surgical procedure, with the number of primary surgeries reaching its highest point in the United Kingdom (UK) at nearly 200,000 before the COVID pandemic (2018) down to 105,000 in 2020 [1]. The British Orthopaedic Association recommended that long-term follow-up visits begin at 5 or 7 years after the primary joint replacement depending on the type of implant, and then continue three-yearly thereafter [2, 3]. The aim of the long-term follow-ups by the orthopaedic team is to screen for asymptomatic changes in the replaced knee or hip, including aseptic loosening or osteolysis [4]. These changes can lead to revision surgery, where implant components may be removed, added or replaced depending on the problem that led to the re-operation [5].

The benefit of this practice for patients, however, remains inconclusive. With hospitals facing rising demand for outpatient services, it is necessary that resources currently made available by healthcare providers and the healthcare system more generally are effectively leading to their intended purposes. This study examined the association between long-term follow-up hospital visits to the orthopaedic department and revision rates for patients who undergo knee or hip replacement surgery in England. Mortality rates were also examined given their competing risk relationship with revision rates [6].

## Methods

### Study design and setting

Primary care data from the Clinical Practice Research Datalink (CPRD) GOLD dataset were linked to hospital admissions records from Hospital Episodes Statistics (HES) data in England, which is managed by NHS Digital [7, 8]. CPRD GOLD is representative of the UK population in terms of age and sex, and for this analysis data were extracted from January 1999 to February 2016. Records about inpatient hospital admissions were provided by HES Admitted Patient Care (HES APC) and records for outpatient hospital visits by HES Outpatient. HES APC contains data on all inpatients’ hospital admissions to the NHS in England, including elective and non-elective admissions, and those data were extracted from 1999 to 2016 [9]. Outpatient appointment records are included in HES Outpatient, and data were extracted from 2004, which is the earliest available date, to 2016. Data on mortality were also retrieved using guidelines to reconcile the information present in CPRD with those in Office for National Statistics (ONS) records [10]. ONS is an independent national department collecting and disseminating a range of economic, population, and social statistics, including mortality statistics.

### Participants

Patients were identified separately for procedures related to primary knee and hip surgery using HES APC records. Relevant procedures were identified in the OPCS-4 classification system for primary knee and hip replacement based on the selection of codes provided by the National Joint Registry (NJR) [11]. Since we aimed to investigate the impact of follow-up visits occurring 5 years after the primary joint replacement, we included only patients with at least 6 years of available data to be able to identify both a potential follow-up consultation and the outcome of interest. During those first 6 years patient records must not report revision, another primary joint surgery or death. Data from patients were retrieved up to 10 years since primary joint replacement to ensure that a substantial number of patients was available for analysis. Furthermore, patients with records for both primary knee and hip replacements were excluded as otherwise we could not identify which joint the follow-up visit was meant to assess. Because HES Outpatient data, which were used to operationalise follow-up visits, are available only from 2004 onwards, we included patients who had a primary joint replacement in or after 1999, so as to ensure that a follow-up visit after 5 years would be recorded.

### Comparator groups

Two cohorts of patients were created, one with those reporting at least one long-term follow-up visit to the outpatient orthopaedic department (‘Follow-up group’), and those without any such visits (‘No follow-up group’). A set of rules to assign patients to the Follow-up group was followed and is described in detail in the Electronic Supplementary Material.

### Index date

A time-dependent covariate approach was used, because follow-up consultations could take place anytime between 5 years after primary operation and the end of the exposure period (i.e. up to 10 years after primary surgery). This approach is used to avoid introducing ‘immortal time bias’, which refers to a period of cohort exposure time during which death (or other outcomes that indicate the end of exposure time) cannot occur [12]. Based on this approach, all patients were included in the No follow-up group at 5 years after the primary replacement until their first long-term follow-up visit was identified, if any, at which point the patient became part of the Follow-up group. Consequently, patients without any follow-up visit remained in the No follow-up group for the entirety of their exposure period. Index date for the purposes of identifying outcomes of interest was therefore 6 years after primary surgery for the No follow-up group, and the date of first follow-up visit to the orthopaedics department for the Follow-up group.

### Outcome measures

We estimated the cumulative incidence for each of the two groups and compared the number of revisions, which indicates the probability of having a revision within the exposure period before the occurrence of the competing risk of death [13]. We also used Cox proportional hazards regression models to estimate cause-specific hazard ratios (HR) and their 95% confidence intervals (CI) for both revisions and mortality.

### Statistical analysis

The clinical and demographic characteristics of patients in the two groups were examined using descriptive statistics and compared using standardised mean differences. For each outcome, exposure time ended at the earliest of: (1) a second knee or hip replacement (i.e. assumed to be contralateral, as data did not provide details for laterality), (2) the 10 year follow-up period, (3) the end of data extraction, (4) revision surgery, or (5) death. Univariable regression models were initially estimated with only the group variable (Follow-up vs No follow-up) included as explanatory variable. Subsequently, multivariate models were estimated including age, sex, year of primary surgery, ethnicity, and Charlson comorbidity index (dichotomized as 0 and 1+ due to relatively small numbers of patients with multiple comorbidities). A detailed description of the variables included in the analysis can be found in the Electronic Supplementary Material. Because we used administrative data required for hospital reimbursement, there was no missing data in any of the variables analysed.

Data analysis was performed in R 3.5.2 using the packages ‘dplyr’ for data manipulation, ‘survival’ for fitting regression models, and ‘ggplot2’ for producing cumulative incidence plots [14–17]. We followed the STROBE statement as a reporting guideline for this study [18].

## Results

### Study participants

**Fig. 1:**
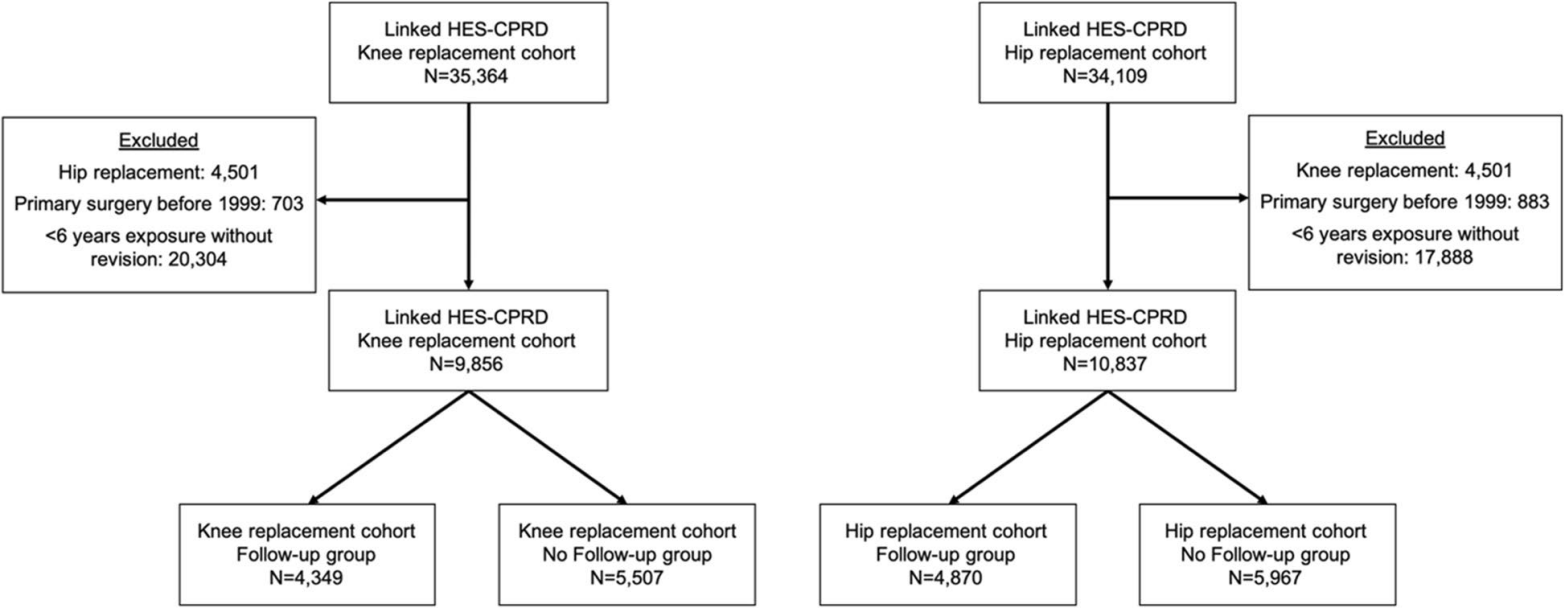
Flowchart describing the inclusion of patients

**Fig. 2:**
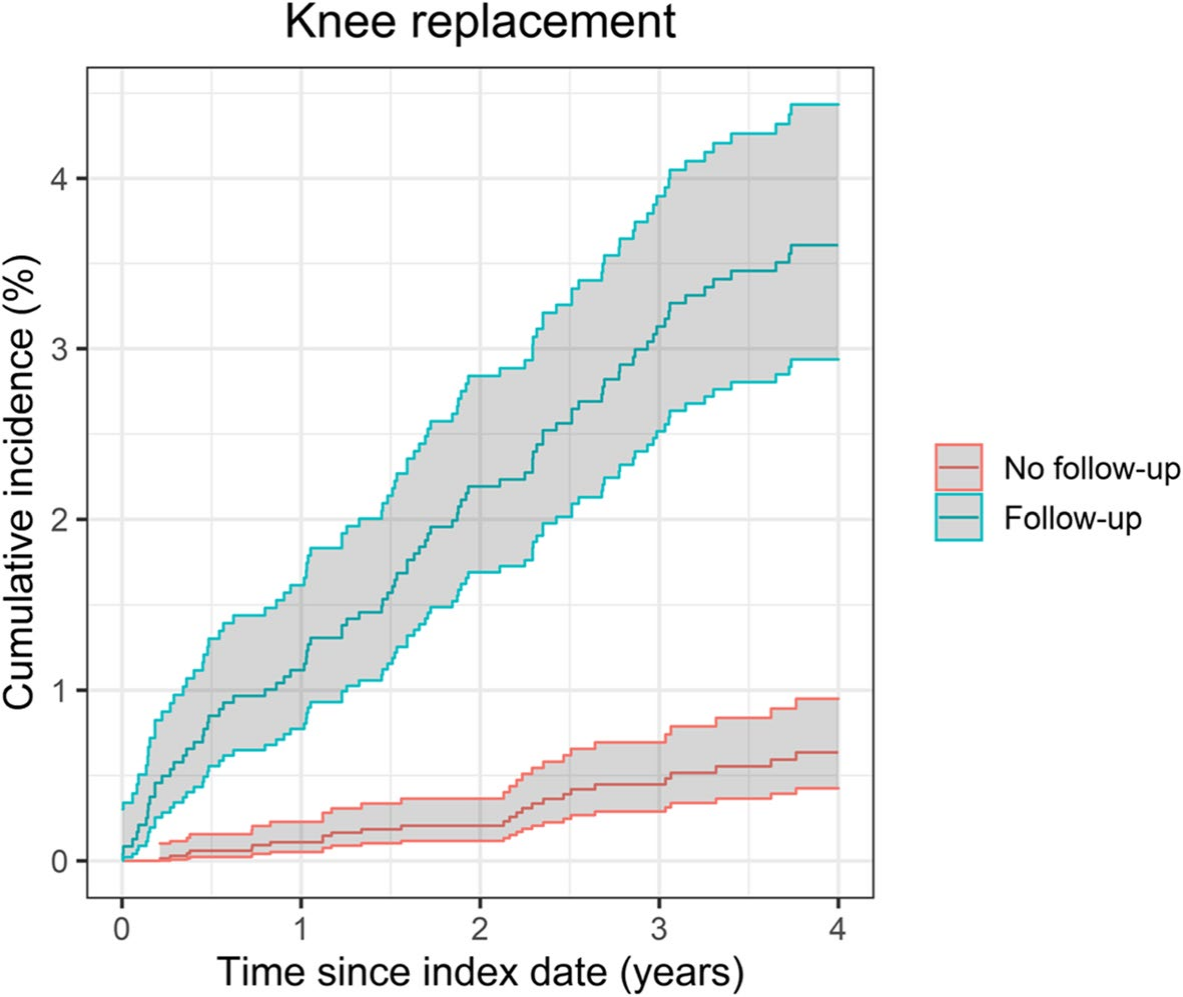
Cumulative incidence of revision following knee replacement (from five years after primary surgery)

**Fig. 3:**
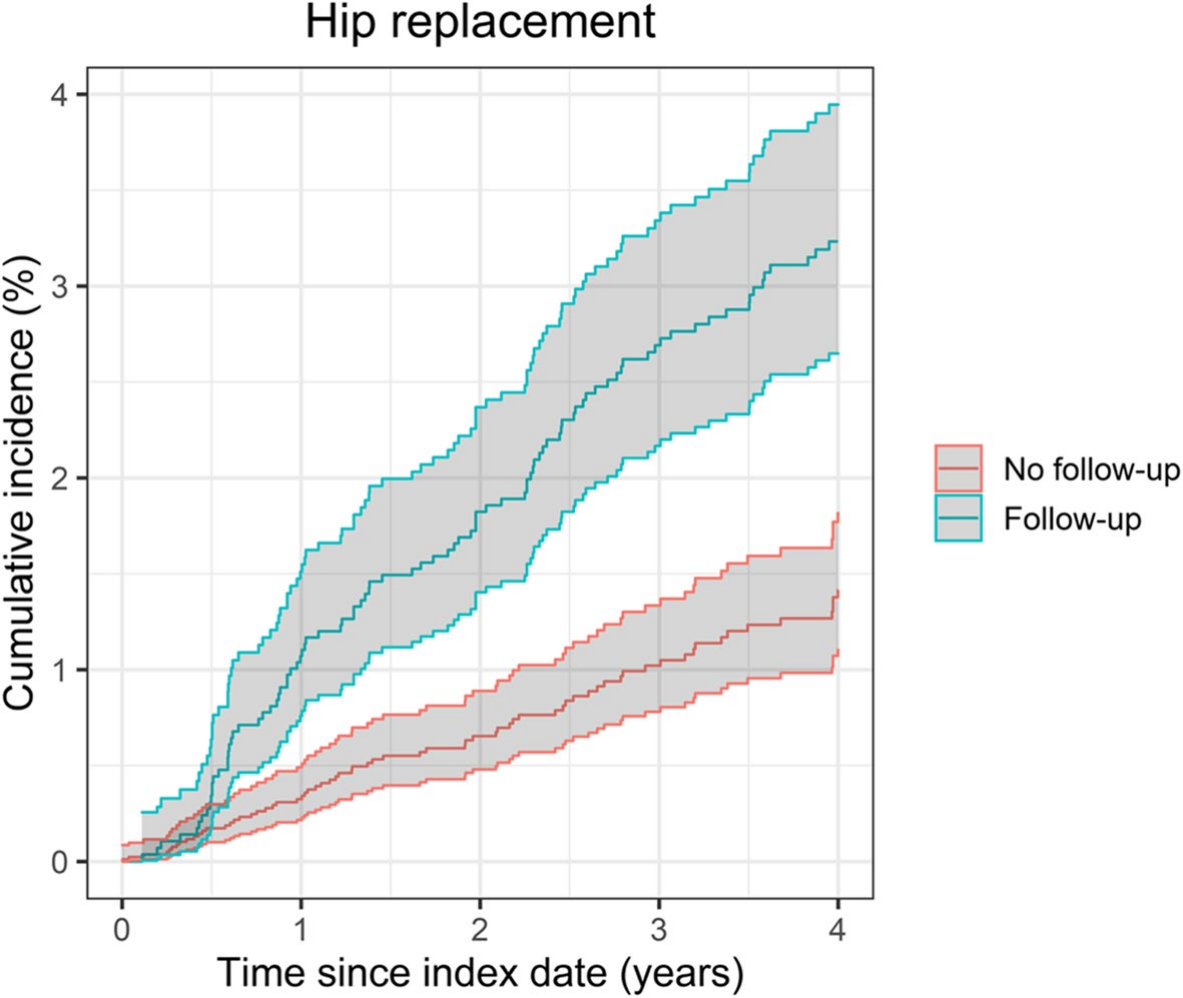
Cumulative incidence of revision following hip replacement (from five years after primary surgery)

The flow of patients’ inclusion is illustrated in Fig. 1 and the clinical and demographic characteristics of those included are detailed in Table 1. The estimate of the cumulative incidence function of the time from primary joint replacement to the first long-term follow-up visit is shown in Fig. S1 in the Electronic Supplementary Material. The number of events and cumulative incidence of revision and mortality are described in Table 2. Figures 2 and 3 show the cumulative incidence of revision for the Follow-up and No follow-up groups, accounting for time-varying exposure and with the index date being 6 years after primary surgery in the case of the No follow-up group and date of first follow-up visit to the orthopaedics department at least 6 years after primary surgery in the case of the Follow-up group. In both cases, the incidence of revision was higher in the Follow-up group compared with the No follow-up group.

**Table 1:** Description of demographic and clinical characteristics of patients

Characteristics	Knee replacement (***n*** = 9856)			Hip replacement (***n*** = 10,837)		
	Follow-up	No follow-up	SMD	Follow-up	No follow-up	SMD
n (%)	4349 (44%)	5507 (56%)	NA	4870 (47%)	5967 (53%)	NA
Sex: Female (n [%])	2601 (60%)	3090 (56%)	0.075	3161 (65%)	3528 (59%)	0.030
Age (median [IQR])	69 (62–75)	72 (65–78)	0.322	67 (60–73)	71 (64–78)	0.401
Ethnicity: White (n [%])	3314 (76%)	4110 (75%)	0.036	3737 (77%)	4372 (73%)	0.080
Year of surgery (median [IQR])	2005 (2003–2007)	2006 (2004–2008)	0.268	2005 (2003–2007)	2006 (2003–2008)	0.220
Follow-up visits (median [IQR])	3 (1–5)	NA	NA	3 (1–4)	NA	NA
Years from primary surgery to first follow-up visit (median [IQR])	5.9 (5.2–6.9)	NA	NA	5.9 (5.2–6.9)	NA	NA
Myocardial infarction (n [%])	12 (0.28%)	20 (0.36%)	< 0.001	16 (0.32%)	30 (0.50%)	< 0.001
Venous thromboembolism (n [%])	45 (1.03%)	64 (1.16%)	< 0.001	68 (1.40%)	78 (1.31%)	< 0.001
Prosthetic joint infection (n [%])	6 (0.14%)	1 (0.02%)	NA	2 (0.04%)	4 (0.07%)	< 0.001
RCS Charlson (n [%])			0.018			0.027
0	3489 (80.2%)	4436 (80.6%)		4083 (83.8%)	4967 (83.2%)	
1	737 (16.9%)	924 (16.8%)		685 (14.1%)	853 (14.3%)	
2	108 (2.5%)	133 (2.4%)		91 (1.9%)	133 (2.2%)	
3+	15 (0.3%)	14 (0.3%)		11 (0.2%)	14 (0.2%)	

*Abbreviations*: *IQR* interquartile range, *n* number, *NA* Not applicable, *RCS* Royal College of Surgeons, *SMD* Standardised mean difference

**Table 2:** Revision and mortality rates at 10 years after primary surgery

	Follow-up		No follow-up	
	Number of events	Cumulative incidence (% [95% CI])^a^	Number of events	Cumulative incidence (% [95% CI])^a^
**Knee replacement**				
Revision	88	3.6% (2.9 to 4.4%)	26	0.6% (0.4 to 1.0%)
Mortality	392	16.8% (15.2 to 18.3%)	900	20.7% (19.4 to 22.1%)
**Hip replacement**				
Revision	94	3.2% (2.7 to 4%)	68	1.4% (1.1 to 1.8%)
Mortality	427	14.9% (13.7 to 16.3%)	1024	21.2% (19.9 to 22.5%)

^a^accounting for time-varying exposure

### Association between long-term follow-up and revision knee replacement

The incidence of revision at 4 years from index date (i.e. 6–10 years since primary knee replacement) was higher for patients in the Follow-up group (3.6%) compared to those in the No follow-up group (0.6%) after allowing for the competing risk of mortality. This difference was statistically significant as indicated by the adjusted regression model (HR: 5.65 [3.62 to 8.81]), (Table 3). Older age at surgery was associated with lower risk of revision (HR: 0.95 [0.93 to 0.96]). The detailed estimates from the multivariable adjusted Cox regression models can be found in Table S1 in the Electronic Supplementary Material.

**Table 3:** Difference in risk for revision and mortality between the Follow-up and No follow-up groups accounting for time-varying exposure

	Hazard ratio (95% CI)	
	Unadjusted model	Multivariable model^a^
**Knee replacement**		
Revision	6.35 (4.408 to 9.86)	5.65 (3.62 to 8.81)
Mortality	0.77 (0.68 to 0.87)	0.95 (0.84 to 1.07)
**Hip replacement**		
Revision	2.34 (1.71 to 3.20)	2.04 (1.48 to 2.81)
Mortality	0.68 (0.61 to 0.76)	0.91 (0.81 to 1.02)

^a^Model adjusted for age, sex (male vs female), RCS Charlson score (0 vs 1 or more), ethnicity (white vs other), and year of surgery

### Association between long-term follow-up and mortality after knee replacement

The incidence of death at 4 years from index date (10 years after surgery) was lower for patients in the Follow-up group (17%) compared to those in the No follow-up group (21%), but this difference was not statistically significant as indicated by the adjusted regression model (HR: 0.95 [0.84 to 1.07]), (Table 3). Older age at surgery (HR: 1.12 [1.11 to 1.13]) and higher RCS Charlson score (HR: 1.58 [1.39 to 1.79]) were associated with higher risk of death. Female sex (HR 0.78 [0.70 to 0.88]) and year of surgery being closer to the end of study’s follow-up time (HR 0.7 [0.94 to 0.99]) were associated with lower risk of death.

### Association between long-term follow-up and revision hip replacement

As with knee replacement, the incidence of revision at 4 years from index date (10 years after surgery) was higher for patients in the Follow-up group (3.2%) compared to those in the No follow-up group (1.4%). Based on the adjusted regression model, this difference was statistically significant (HR: 2.04 [1.48 to 2.81]), (Table 3). Older age at surgery was associated with lower risk of revision (HR: 0.97 [0.96 to 0.98]).

### Association between long-term follow-up and mortality after hip replacement

The incidence of death at 4 years from index date was also lower for patients in the Follow-up group (15%) compared to those in the No follow-up group (21%), but this difference was not statistically significant as indicated by the adjusted regression model (HR: 0.91 [0.81 to 1.02]), (Table 3). Older age at surgery (HR: 1.10 [1.10 to 1.11]) and higher Charlson score (HR: 1.41 [1.24 to 1.61]) again were associated with higher risk of death, whilst female sex (HR 0.81 [0.73 to 0.90]) was associated with lower risk of death.

## Discussion

This study examined the association between hospital visits to the orthopaedic department and revision as well as mortality rates up to 10 years after primary surgery for patients who underwent knee or hip replacement surgery. We found higher risk for revision for the Follow-up group compared to the No follow-up group for both knee and hip replacement. This difference was statistically significant after controlling for age, sex, year of primary surgery, ethnicity, and Charlson score. For both joints, there were no statistically significant differences in mortality rates between the two groups.

The clinical and demographic characteristics of patients in the two groups were largely similar. This is in line with a previous study which found no statistically significant differences in the characteristics of those attending and those not attending follow-up visits after knee replacement surgery [19]. However, we found that patients in the Follow-up group were younger compared with No follow-up group patients. It is possible that older patients are less likely to benefit from prophylactic revision to prevent further damage in the replaced joint later in life, and this may lead to these patients not being invited for follow-up hospital visits.

The fact that less than half of the patients had a record of a long-term follow-up visit in the orthopaedic department is not in accordance with the clinical guidelines that recommend follow-up visits for all patients with knee and hip replacement. Our findings indicate that recommendations from clinical guidelines are not always followed, but this can be explained by the variation in clinical practice. In particular, a previous study showed that follow-up after 5 years since primary hip surgery is offered only by 43% of hospital units in the UK [4]. The number and type of follow-up visit offered to patients with joint replacements as part of the monitoring protocol followed by hospital units should be explored in future studies.

Just over half of patients included in this study did not have any follow-up visits, with similar proportions in the knee and hip cohorts. A common explanation from patients not attending long-term follow-up visits is that they do not have any problems with the replaced joint [19]. Based on our findings, however, a few patients in the No follow-up group did have a record of revision; although those events were rare (0.6 and 1.4% for knee and hip, respectively). This may reflect an unmet need for more revision surgeries in this group. Patient-reported outcomes, including pain, function, and health-related quality-of-life are useful indicators of problems linked to knee and hip replacements. Nevertheless, the joint assessment from the specialised orthopaedic team during a follow-up visit remains essential for the identification of degenerative changes, which sometimes can be asymptomatic and not reflected in patient-reported outcome alone [20, 21].

### Strengths and limitations

This study has important limitations worth mentioning. A critical aspect of this study was the identification of records of long-term follow-up visits. We used the code ‘110 = Trauma and Orthopaedics’ to identify such visits, but it was not possible to know the reason for each visit, the actual scope of the consultation, or if each recorded visit was directly associated with the replaced joint. Not knowing the reason for the outpatient consultation or whether it was routine or not could have a significant impact on the results and interpretation of results. This could only be collected via a prospective study which is not what this study used for the analysis; it would however be important for future studies to collect these data and conduct a similar analysis with a clear understanding of which outpatient consultations were planned, and which were not. To mitigate this problem as much as possible considering the limitations of the dataset we used, a group of rules to define follow-up visits was set, as elaborated in the Electronic Supplementary Material, to increase our confidence that the identified visits at the very least regarded the knee or hip replacement. Furthermore, primary care referral records from CPRD GOLD were used to validate the follow-up visits identified in HES Outpatient. We found that visits to the orthopaedic department as recorded in HES Outpatient were mostly reported in CPRD.

The study was also restricted by other limitations inherent to the dataset used. The NHS Hospital Episodes Statistics does not capture details that can potentially play a relevant role in predicting revision such as implant model, type of prosthesis, surgical approach, surgeon preferences and experience, and critically reason for revision, to name a few. Regarding the reason for revision, as the aim of our analysis was precisely to examine the association between long-term follow-up and incidence revision, not having access to details about why revisions were undertaken limit the implications of our findings. For example, revisions for dislocation and infection would tend to present acutely and hence not be connected to long-term follow-up. This was not a problem in our analysis as we captured the outcome of revision only after 5 years had passed since the primary surgery, thus eliminating the possibility that future follow-up visits could impact earlier revisions. Notwithstanding this, the point remains that reason for revision should be included in any analysis of this sort so that only those revisions that could be impacted by long-term follow-up are included in the analysis. Similarly, the outcome of revision would be impacted by the decision-making process that individual surgeons follow when deciding on whom and when to conduct a primary or revision arthroplasty. As a factor having such a relevant weight on the incidence of the outcome, it should be included in studies like this one. Because we used data from the NHS Hospital Episodes Statistics, we could not include this variable, however future studies could access data from the National Joint Registry where details on the operating surgeon are available, as is the reason for revision. Further, our analysis was performed only up to 10 years after primary due to lack of sufficient data beyond that point, would have potentially affected findings. It would be important for further studies to be conducted with more comprehensive datasets such as the same NHS HES but linked to the National Joint Registry records, which include many relevant surgical details, and at a point when sufficient data is available for a period of 15–20 years after primary arthroplasty.

We cannot establish a causal association between follow-up visits and revision rates in our study. It is possible that patients that received follow-up were different from patients that did not attend these visits in terms of clinical characteristics and healthcare needs. Causal association between follow-up visits and longer term outcomes could be established by randomised controlled trials or studies using observational designs applying statistical methods, such as propensity score matching, to minimise the risk of confounding by indication [22]. Finally, more work is needed from future studies to identify the impact of follow-up visits into the second decade following the joint replacement, as well as to explore whether these results might be generalisable to other settings based on different health seeking behaviours and offer of healthcare services in other countries.

This study, however, has also a number of important strengths. Firstly, this is one of the first studies investigating the impact of follow-up after knee and hip replacement using real-world, routinely-collected data from a large cohort of patients who have actually used NHS services in recent years. Secondly, we used reliable inpatient hospital data linked to primary care records to identify patients with primary and revision surgery, as well as outpatient hospital data to determine if the patients had follow-up visits. Finally, the exposure time used in the analysis of 10 years following primary surgery is unlikely to be replicated by other study designs such as randomised controlled trials.

### Association between follow-up and revision knee and hip replacement

The higher revision rates for patients in the Follow-up group for both joints could be an indication of the effectiveness of follow-up visits as a monitoring tool for patients in need of revision surgery. Timely identification of patients with deteriorating implants can prevent considerable bone loss, aseptic loosening, or osteolysis [4, 23]. However, it is possible that these signs are less frequent in the mid-term period between six and 10 years after primary surgery, and studies with longer follow-ups are required. A likely explanation for the higher risk for revision in the Follow-up group is that surgeons are effective arbitrators of the selection of patients to be placed in follow-up lists. Whilst general clinical guidelines are variably adhered to, the final judgement as to which patients are placed in the long-term follow-up pathway is made by surgeons. As such, if they assess a patient as being likely to see their prosthesis fail, then they will mark that patient for long-term follow-up. On the other hand, if the surgeon feels that a patient is unlikely to cause failure to their prosthesis, then that patient is more likely to be placed in the no follow-up group and discharged. The follow-up group would hence be comprised of patients more likely to need a revision, some of which might be prevented with the follow-up, some others requiring the revision, as identified in this study. It should be highlighted that only a small proportion of patients having long-term follow-up eventually had a revision within the period of study (3–4%), but revision rates after 10 years were not identified in this study.

It is also important to examine if the additional costs associated with the follow-up outpatient visits lead to improved patient-reported outcomes and decreased costs of a subsequent revision. The possibility of overtreating patients with joint replacements has been discussed in previous studies [24–26]. Overtreatment may have an impact on the increased revision rates observed for the Follow-up group in our study. Conclusive evidence of overtreatment of patients with joint replacements, however, is still lacking. Future studies should investigate potential overtreatment of patients having long-term follow-up visits, whether it negatively impacts patients’ wellbeing, and if it leads to increased costs for the healthcare systems.

In our analyses, older age was associated with lower risk for revision. This is in line with the results from previous studies that have also found a decreased revision risk for older patients, which can be explained by the more complicated health problems of this population and the increased risk of adverse events linked to revision surgery [5, 27]. Other patients’ characteristics were not associated with revision risk in our study. Older age, comorbidities, and male sex were associated with higher risk of death in the multivariate models for both joints, which has also been reported previously [28].

### Association between follow-up and mortality after knee and hip replacement

The lack of statistically significant differences in mortality rates between those who attended hospital orthopaedic follow-up consultations and those who did not was expected as there is no evident connection between the two. Mortality is, however, naturally linked with age and comorbidities (examined in the model via the RCS Charlson comorbidity index), and these, along with being a man, were identified in the knee and hip models as being associated with higher mortality. After adjusting for these variables, our findings suggest that attending hospital orthopaedic follow-up consultations has no impact on mortality.

## Conclusions

Patients who attended outpatient follow-up consultations up to 10 years after primary joint replacement had higher risk for revision compared with patients who did not. This is potentially a reflection of the effective judgement made by surgeons when they select patients to be included in follow-up lists. Only a small percentage of patients in the Follow-up group actually had a revision. However, findings are subject to important limitations such as the lack of details about reason for outpatient consultation, reason for revision, and surgeon criteria for revision, all of which can potentially play a crucial role in the association examined. Future studies should investigate if the additional cost associated with the outpatient visits lead to improved outcomes and decreased costs of a subsequent revision.

## Supplementary Information

**Additional file 1: Fig. S1.** Cumulative incidence of first long-term follow-up visit to the orthopaedic department in years since primary surgery. **Table S1.** Adjusted regression models for revision and mortality.

## Data Availability

Aggregate summaries of the data that support the findings of this study are available from the corresponding author but restrictions apply to the availability of these data, which were used under license for the current study, and so are not publicly available. Specifically, the patient-level datasets analysed during the current study are subject to restrictions specified under the agreement governing the use of the anonymized data approved specifically for the purpose of this study by the ISAC MHRA Database Research and are hence not publicly available. Data were securely held and analyses conducted at the University of Oxford.

## Abbreviations

APC: Admitted patient care
CI: Confidence interval
CPRD: Clinical Practice Research Datalink
HES: English Hospital Episodes Statistics
HR: Hazard ratio
NJR: National Joint Registry
ONS: Office for National Statistics
RCS: Royal College of Surgeons
