# Supplementary material for: Association between outpatient follow-up and incidence of revision after knee and hip replacements: a population-based cohort study
Source: BMC Musculoskelet Disord. 2023 Feb 8;24:106. doi: 10.1186/s12891-023-06190-7 (PMC9906879; doi:10.1186/s12891-023-06190-7)
Supplement: Supplementary file 1 — Additional file 1: Fig. S1. Cumulative incidence of first long-term follow-up visit to the orthopaedic department in years since primary surgery. Table S1. Adjusted regression models for revision and mortality. [file 12891_2023_6190_MOESM1_ESM.docx]

# Electronic Supplementary Material

## Comparator groups

Only attended visits were considered follow-up and those missed not considered for patient allocation to groups. The rules to assign patients in the Follow-up group were the following: i) the patient must have at least one HES Outpatient record with the code ‘110 = Trauma and Orthopaedics’, describing the specialised service within which the patient was treated, at least five years after primary surgery [5]; ii) in case a record for revision surgery was identified, the follow-up visits occurring within the waiting period (i.e. the time between the date of the decision to have revision surgery and the actual date of surgery) did not count as follow-up; instead, we assumed that those visits were a consequence of the decision to have the operation (e.g. prepare for surgery); and iii) when patients had only a single follow-up visit within six months before the date of the decision to have revision surgery, the visit was assumed to be linked to the decision for revision surgery (e.g. blood tests, MRIs, check-ups for cardiovascular problems) than part of routine long-term follow-up visits and was hence not counted as follow-up. The patients not included in the Follow-up group were assigned to the No follow-up group.

## Variables

We used the OPCS-4 procedures in HES APC records to identify revisions, similar to the procedure described above for the identification of primary joint replacement [4]. The ethnicity of patients (white or other) was recorded in HES APC. The age at time of surgery was calculated using the date of the procedure recorded in HES APC combined with the patient’s year of birth from CPRD GOLD. The patients’ sex were recorded in CPRD GOLD, and we also calculated the Charlson score using ICD-10 diagnosis codes in HES APC. Charlson score is a measure of comorbidity, and the overall summary score can take the values 0, 1, 2, or 3+ [1]. Using data from HES APC we identified three types of complications within 90 days following primary knee or hip surgery. Myocardial infarction (MI) and venous thromboembolism (VTE) were identified using the primary ICD diagnosis code of a hospital episode (i.e. a period of hospital care under one consultant), which occurred the day of the primary joint replacement or within the following 90 days [2]. Prosthetic joint infection (PJI) was identified by the primary ICD diagnosis code of a hospital episode and a concurrent or subsequent operation (OPCS-4) code of either debridement, antibiotics, irrigation, or retention of the prosthesis within a year of the diagnosis [3]. The outcomes of interest, i.e. revision surgery and death, were identified after year six post primary as any such event between years five and six would have resulted in the patient being excluded.

Figure S1. Cumulative incidence of first long-term follow-up visit to the orthopaedic department in years since primary surgery

a) Knee replacement


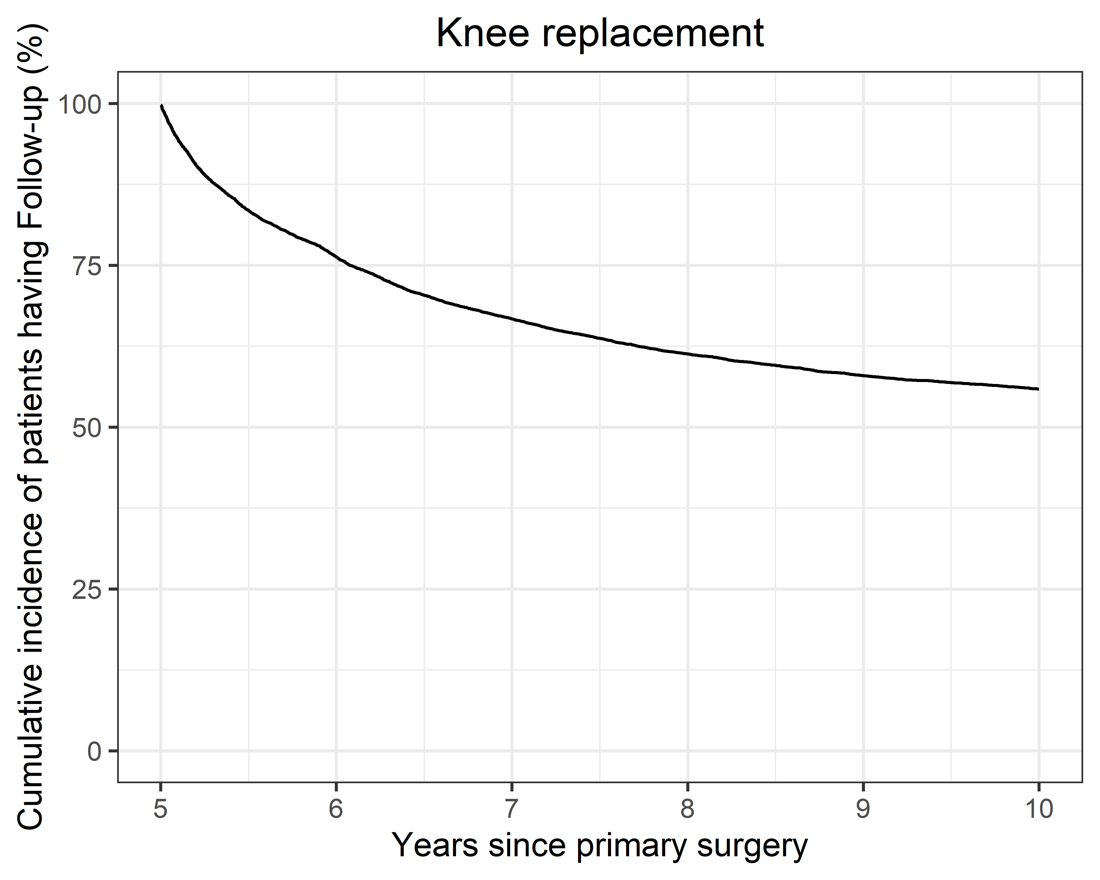


b) Hip replacement


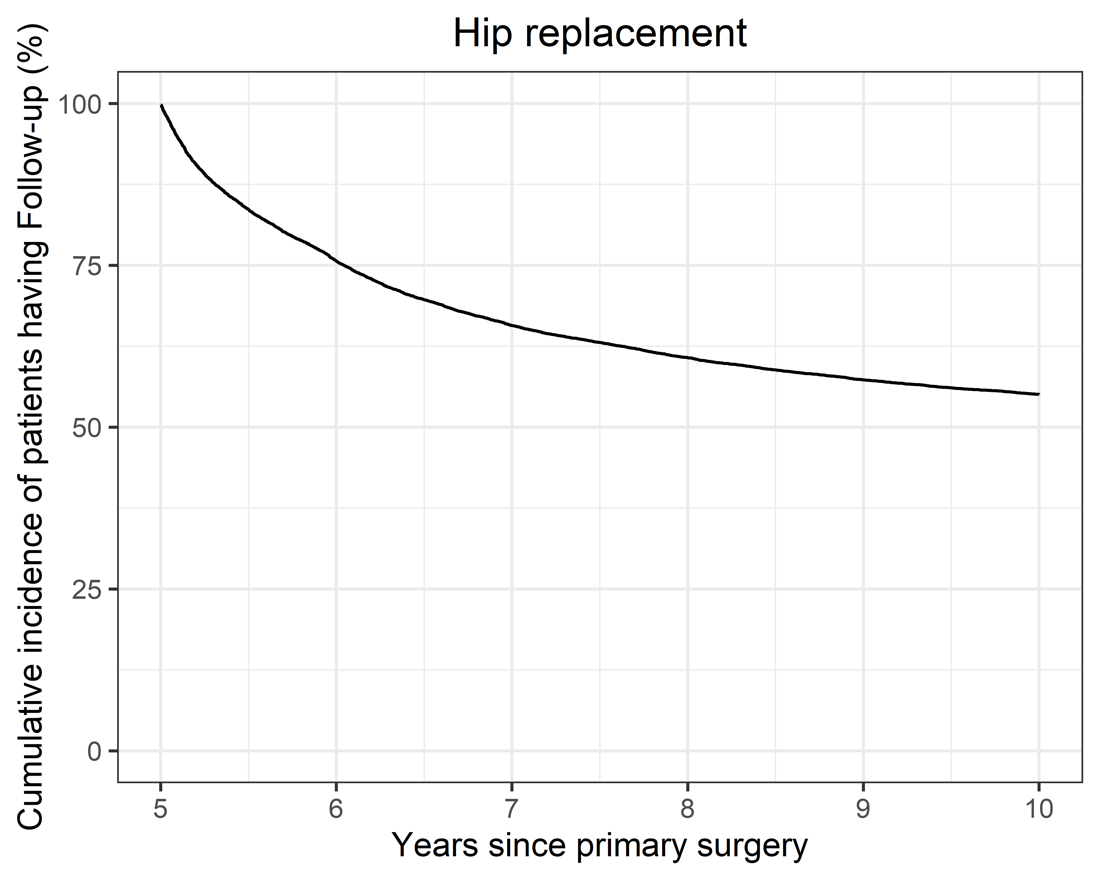


**Table S1.** Adjusted regression models for revision and mortality

|  | **Estimated effect of Follow-up compared to No follow-up, hazard ratio (95% CI)*** | |
| --- | --- | --- |
|  | **Knee replacement** | **Hip replacement** |
|  | Hazard ratio (95% CI) | Hazard ratio (95% CI) |
| *Revision* |  |  |
| Follow-up group | 5.65 (3.62 to 8.81) | 2.04 (1.48 to 2.81) |
| Age at primary surgery | 0.95 (0.93 to 0.96) | 0.97 (0.96 to 0.98) |
| Sex (female) | 1.34 (0.92 to 1.93) | 1.26 (0.91 to 1.75) |
| Charlson score (>=2) | 0.60 (0.35 to 1.07) | 0.97 (0.62 to 1.51) |
| Ethnicity (other than white) | 1.12 (0.75 to 1.69) | 0.99 (0.70 to 1.43) |
| Year of surgery | 0.98 (0.91 to 1.05) | 1.03 (0.97 to 1.09) |
| *Mortality* |  |  |
| Follow-up group | 0.95 (0.84 to 1.07) | 0.91 (0.81 to 1.02) |
| Age at primary surgery | 1.12 (1.11 to 1.13) | 1.10 (1.10 to 1.11) |
| Sex (female) | 0.78 (0.70 to 0.88) | 0.81 (0.73 to 0.90) |
| Charlson score (>=2) | 1.58 (1.39 to 1.79) | 1.41 (1.24 to 1.61) |
| Ethnicity (other than white) | 1.00 (0.87 to 1.13) | 0.91 (0.80 to 1.02) |
| Year of surgery | 0.97 (0.94 to 0.99) | 0.99 (0.97 to 1.01) |

* accounting for time-varying exposure

**References**

1. Armitage JN, van der Meulen JH. Identifying co-morbidity in surgical patients using administrative data with the Royal College of Surgeons Charlson Score. Br J Surg. 2010;97:772-781.

2. Burn E, Edwards CJ, Murray DW, Silman A, Cooper C, Arden NK, Prieto-Alhambra D, Pinedo-Villanueva R. The impact of rheumatoid arthritis on the risk of adverse events following joint replacement: a real-world cohort study. Clinical Epidemiology. 2018;10:697-704.

3. Graves N, Wloch C, Wilson J, Barnett A, Sutton A, Cooper N, Merollini K, McCreanor V, Cheng Q, Burn E, Lamagni T, Charlett A. A cost-effectiveness modelling study of strategies to reduce risk of infection following primary hip replacement based on a systematic review. Health Technol Assess. 2016;20:1-144.

4. OPCS Codes relevant to procedures recorded on the NJR, 2016.

5. HES Data Dictionary Outpatients, 2018.
